# Separate *Polycomb Response Elements* control chromatin state and activation of the *vestigial* gene

**DOI:** 10.1371/journal.pgen.1007877

**Published:** 2019-08-19

**Authors:** Kami Ahmad, Amy E. Spens

**Affiliations:** Division of Basic Sciences, FHCRC, Seattle, WA, United States of America; Centre National de la Recherche Scientifique, FRANCE

## Abstract

Patterned expression of many developmental genes is specified by transcription factor gene expression, but is thought to be refined by chromatin-mediated repression. Regulatory DNA sequences called *Polycomb Response Elements* (*PRE*s) are required to repress some developmental target genes, and are widespread in genomes, suggesting that they broadly affect developmental programs. While *PRE*s in transgenes can nucleate trimethylation on lysine 27 of the histone H3 tail (H3K27me3), none have been demonstrated to be necessary at endogenous chromatin domains. This failure is thought to be due to the fact that most endogenous H3K27me3 domains contain many *PRE*s, and individual *PRE*s may be redundant. In contrast to these ideas, we show here that *PRE*s near the wing selector gene *vestigial* have distinctive roles at their endogenous locus, even though both *PRE*s are repressors in transgenes. First, a *PRE* near the promoter is required for *vestigial* activation and not for repression. Second, only the distal *PRE* contributes to H3K27me3, but even removal of both *PRE*s does not eliminate H3K27me3 across the *vestigial* domain. Thus, endogenous chromatin domains appear to be intrinsically marked by H3K27me3, and *PRE*s appear required to enhance this chromatin modification to high levels at inactive genes.

## Author summary

Eukaryotic genes are packaged in chromatin, and their transcription relies on activators that recruit RNA polymerases and on repressive factors. In multicellular organisms, cell types have distinct patterns of gene expression, and these patterns are controlled by by the expression of cell-type-specific transcription factors and by modulating chromatin structure. The Polycomb system is one major system for the chromatin-mediated silencing of developmental gene expression, where a histone methyltransferase marks extended chromatin domains with trimethylation of lysine-27 of the histone H3 tail (H3K27me3) and forms repressed chromatin. In Drosophila, repressive regulatory elements called *Polycomb Response Elements* (*PRE*s) are thought to nucleate histone methyltransferase binding which then spreads across these domains. In this study, we demonstrate that two *PRE*s near the developmental *vestigial* gene have distinct and separable effects on gene activation and chromatin structure. Both *PRE*s are functional repressors in transgenes, but the *PRE* located near the *vestigial* promoter is required for gene transcription. This *PRE* has no effect on histone methylation of the domain. The second *PRE* located in the middle of the chromatin domain is required for high-level H3K27me3 of the domain, but this methylation is not required to refine *vestigial* gene expression. Significant chromatin methylation remains when both *PRE*s are deleted. Our findings imply that *PRE*s near promoters may play activating roles in gene expression in the Drosophila genome. We suggest that some domains of H3K27me3 may have little consequence for correctly patterning gene expression.

## Introduction

The patterns of chromatin histone modifications differ between cell types, reflecting the activity of genes for developmental programs. Tri-methylation of the lysine-27 residue of histone H3 (H3K27me3) typically marks extended chromatin domains, leading to chromatin compaction and epigenetic gene silencing that is maintained as cells differentiate [1,2]. Histone methylation is thought to be initiated at discrete regulatory elements called *Polycomb Response Elements* (*PRE*s) within domains. These elements bind multiple DNA-binding factors, recruiting the PRC1 and PRC2 complexes, including the Polycomb chromatin factor and the E(z) histone methyltransferase, respectively [3,4]. Transgenes carrying *PRE*s are sufficient to silence reporter genes and to nucleate new H3K27me3 domains [5–7]. However, the function of *PRE*s in their endogenous domains is less clear. Deletion of *PRE*s from the homeobox gene cluster *BX-C* have limited defects in gene silencing [8–10], but no reduction of histone methylation of this domain. While multiple *PRE*s within the *BX-C* domain may be redundant, deletion of all mapped *PRE*s near the *invected* and *engrailed* genes have no effect on methylation of the locus, and it remains unknown how histone methylation is maintained [11].

Genomic mapping has identified regions where both the PRC1 and PRC2 complexes colocalize, and regions where each complex is found separately. Only about one-half of all Polycomb binding sites are within H3K27me3 domains, and thousands of additional sites are located near the promoters of active genes [12,13], where they may modulate gene expression by holding RNAPII at paused promoters. Here, we characterize the *in vivo* roles of two *PRE*s near the *vestigial* gene. While these two *PRE*s are silencers in transgene assays, targeted mutations reveal that the promoter *PRE* is required for full gene expression. Using a new efficient method for genomic mapping of chromatin factors, we demonstrate that methylation across the domain remains in the absence of both *PRE*s. Our results reveal that *PRE*s stimulate but are not necessary for domain methylation.

## Results

### Profiling domains and regulatory elements in dissected tissues

To profile chromatin domains in different tissues, we used a chromatin mapping strategy that tethers micrococcal nuclease at factor binding sites. In the CUT&RUN procedure [14], unfixed cells are soaked with a factor-specific antibody, which binds to chromatin. Next, a protein-A-micrococcal nuclease (pA-MNase) fusion protein is soaked in, binding to the chromatin-bound antibody. Activation of the tethered MNase by adding calcium then cleaves exposed DNA around the binding sites of the targeted factor. Sequencing of the cleaved DNA fragments thus maps the location of the targeted chromatin protein. CUT&RUN obviates the need to work with chromatin preparations or to optimize affinity recovery of chromatin particles, and works efficiently with small numbers of cells [15,16]. To implement CUT&RUN for tissue samples, we simply dissected brains and wing imaginal discs from ten larvae, lightly permeabilized the whole tissues with digitonin, and sequentially incubated the tissues with antibody to H3K27me3 and then with pA-MNase. MNase was then activated and finally the cleaved DNA was isolated, subjected to Illumina paired-end sequencing, and mapped to the Drosophila dm6 genome assembly. We similarly mapped the Polycomb protein, which binds at *Polycomb Response Elements* (*PRE*s).

**Fig 1:**
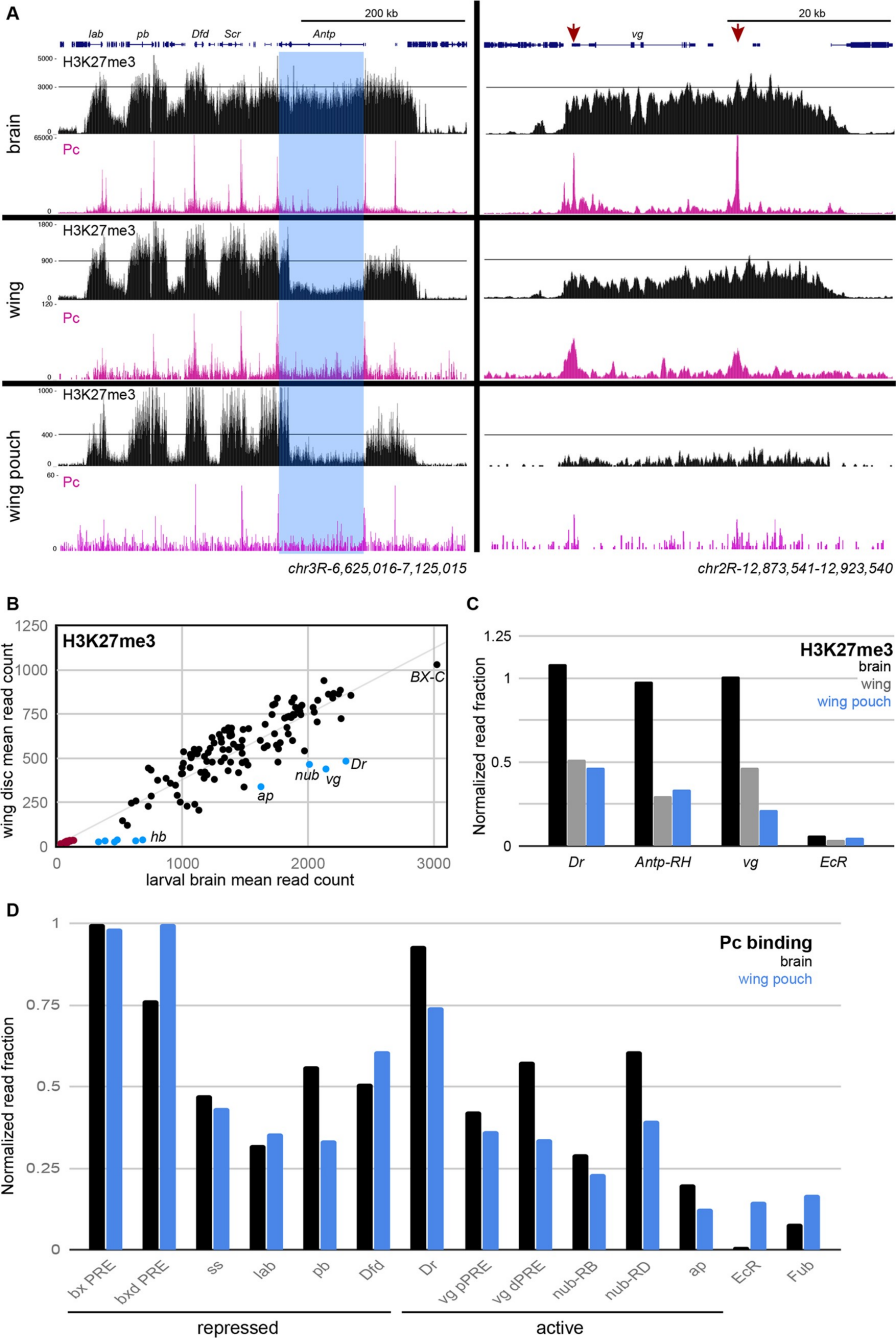
CUT&RUN profiling of Polycomb silenced domains in larval brains and wing imaginal discs. (A) Chromatin landscapes of the 425 kb *ANTP-Complex* (left) and for the 32 kb *vg* domain (right) in dissected larval brains, in dissected wing imaginal discs, and in FACS-isolated wing disc pouch cells. Landscapes for H3K27me3 (black) and Polycomb (magenta) are shown. The *Antp* gene is highlighted in blue; this gene is silent and packaged with high levels of H3K27me3 in brain samples and transcribed and packaged with low levels of H3K27me3 in wing imaginal discs. The two *PRE*s in the *vg* domain are indicated (red arrows). (B) H3K27-trimethylation of domains in larval brains and wing discs. The average read count in similar domains (black) and in domains that are reduced in wing discs (blue) are plotted. Randomly-selected regions outside of domains (red) indicate the genomic background in each sample. (C) Read counts for H3K27me3 CUT&RUN across three genes silenced in larval brains and expressed in wing discs. The *EcR* is not included in an H3K27me3 domain in either tissue, and is a control for the genomic background in each sample. Mean read counts in each domain were normalized by dividing by the mean read counts at the *gsb* gene, which is silenced in all tissues. (D) Mean read counts for Polycomb CUT&RUN at selected repressed peaks in brain and wing pouch cells and at active genes defined by loss of H3K27me3 domains. The *EcR* promoter and the *Fub* insulator element are not bound by Polycomb, and are controls for the genomic background in each sample.

H3K27me3 domains in larval tissues have been previously mapped by Chromatin Immunoprecipitation [17]. Profiles of H3K27me3 distribution generated by CUT&RUN using substantially less material were similar (Pearson’s *r* = 0.94). Both methods reveal changes in chromatin methylation that correspond to tissue-specific changes in gene expression. For example, the *ANTENNAPEDIA-COMPLEX* (*ANTP-C*) cluster of homeobox genes are encompassed in a H3K27me3 domain in larval brains, consistent with the predominant silencing of this cluster in this tissue [18] (**Fig 1A**). In contrast, in wing imaginal discs where *Antp* is transcribed, chromatin over most of this gene is depleted for H3K27me3. Interestingly, some histone methylation remains across the 3’ exons of *Antp*, which indicate that a shorter isoform of the *Antp* gene may be transcribed in this tissue. Notably, histone methylation is not completely eliminated from the transcribed *Antp* gene (**Fig 1A**).

To quantify changes in chromatin landscapes between tissues, we measured the read count coverage at 125 chromatin domains (listed in **S3 Table**) with high H3K27me3 in larval brain and wing disc samples. Most chromatin domains are similarly methylated between these tissues, but a small number of domains have lower read counts for H3K27me3 in wing discs compared to larval brains (**Fig 1B**). One group of domains have low levels of histone methylation in larval brains and lose methylation in wing discs, however, genes in these domains are not expressed in either tissue. A second set of domains encompass genes that are expressed in wing discs but not in brains, including *apterous* (*ap*), *nubbin* (*nub*), *vestigial* (*vg*), and *Drop* (*Dr*) (**Fig 1B**, blue), and histone methylation across these domains is lower in wing discs. Histone methylation is not eliminated, as 25–50% of H3K27me3 levels remains even when domain genes are transcribed, and this is noticeably greater than background levels at random regions outside of domains (red in **Fig 1B, Fig 1C**). Thus, activation of these genes is accompanied by reduction–but not loss–of the H3K27me3 modification.

### The *vestigial* domain contains two *Polycomb response elements*

We focused on the *vg* gene locus as a simple model. The *vg* gene is required for wing determination, is the only gene in a 32 kb H3K27me3 chromatin domain (**Fig 1A**). The *vg* gene is inactive in brain tissues, and is expressed only in the pouch of wing imaginal discs. This domain is heavily methylated in larval brains, but reduced to ~50% across the domain in wing discs (**Fig 1A and 1C**). This is consistent with loss of H3K27me3 when the *vg* gene is activated, but wing discs are a mixture of cells with and without *vg* expression. We therefore isolated *vg*-expressing cells to profile the chromatin status of the active gene. We made a transgene construct containing the *vg* Quadrant enhancer [19], and the *GAL4* transcriptional activator, and used this to drive expression of GFP in the wing pouch. We then used FACS to isolate GFP-positive cells and profiled these cells by CUT&RUN. As expected, profiles of these cells show reduced histone methylation across wing-specific genes like *Antp* (**Fig 1A, Supplementary Information**). We found that H3K27me3 across the *vg* domain is reduced to ~20% of it’s levels in non-expressing cells, but remains ~4-fold more methylated than background levels across the genome (**Fig 1A and 1C**). Thus, while gene activation is associated with elimination of the H3K27me3 modification, a small amount of methylation remains across many activated domains.

**Fig 2:**
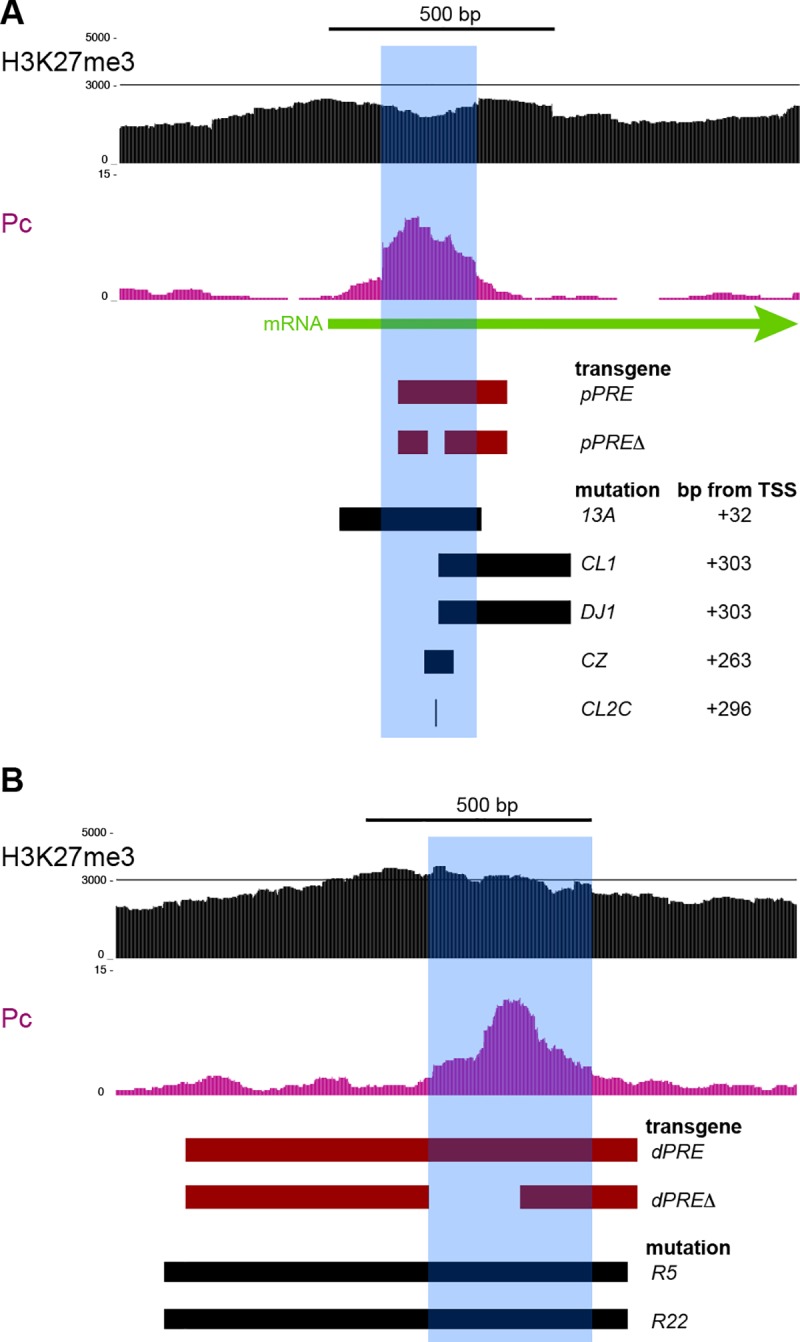
Transgenes and mutations of the two *PRE*s in the *vestigial* domain. H3K27me3 and Polycomb features of the two *PRE*s (blue shading). Segments included in transgene constructs (red), and removed by mutation of the endogenous locus (black) are indicated. (A) The *pPRE* Polycomb-bound site is located from +300 –+400 bp downstream of the transcriptional start site (TSS). The distance of the left end of each deletion from the TSS at the endogenous locus is listed. The *vg*^*CL2C*^ allele is a C->T substitution within the major Polycomb-bound site (chr2R:12,884497 dm6). (B) A segment around the *distal PRE* (*dPRE*) with one Polycomb-bound site. Two engineered deletions (the *vg*^*R5*^ and *vg*^*R22*^ alleles) remove the site.

Two potential *PRE*s within the *vg* domain have been identified by sequence motifs [20] and by chromatin profiling [12,13,21]. The first region, which we term the *proximal PRE* (*pPRE*), is located 300 bp downstream of the mapped Transcriptional Start Site (TSS) of the *vg* gene, which was previously mapped by primer extension [22]. The second *distal PRE* (*dPRE*) is located ~25 kb downstream (**Fig 2A**). We used CUT&RUN to map the Polycomb chromatin protein in larval tissues, and found that this protein is bound at both of these *PRE*s in larval brains (**Fig 1A**). Surprisingly, Polycomb binding is detectable at both *PRE*s in both wing discs and even in FACS-isolated *vg*-expressing cells (**Fig 1A**). We quantified the amount of Polycomb and found that Polycomb is retained to similar levels at the *pPRE* of the active *vg* gene, and at reduced levels at the *vg dPRE* (**Fig 1D**). These levels are clearly above background levels at a non-target promoter or an insulator element. We also observed retention of Polycomb at the promoters of the *Dr* and *nub* genes, which are active in wing pouch cells (**Fig 1D**). The promoter of the *ap* gene which changes methylation between brain and wing disc samples has very low levels of Polycomb in repressed brain samples, and is indistinguishable from background in wing pouch cells (**Fig 1D**). These results indicate that in some cases Polycomb remains present at de-repressed genes.

**Fig 3:**
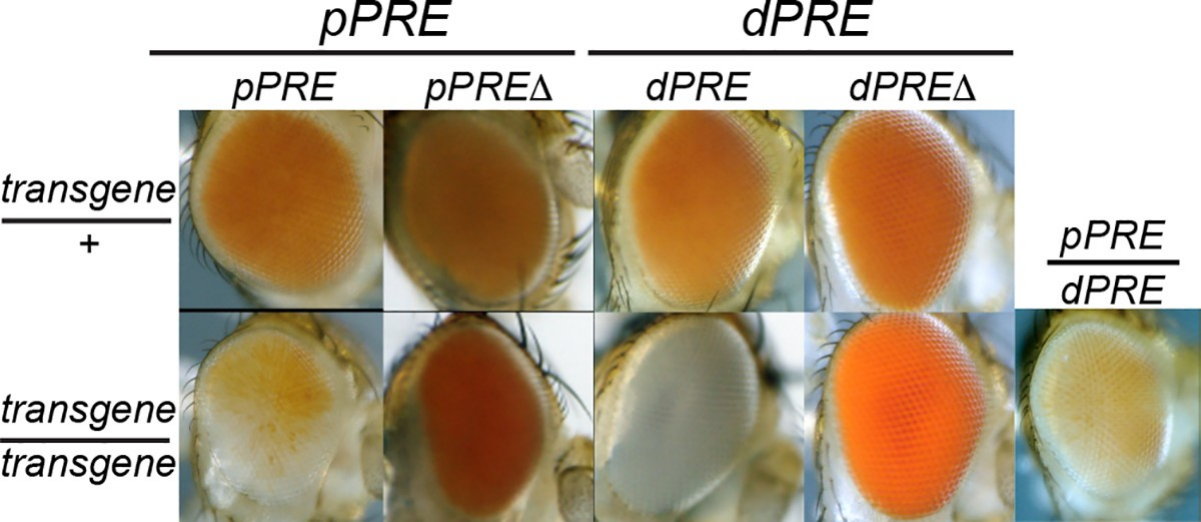
The *vestigial PRE*s mediate silencing of reporter genes. Position-Sensitive silencing (PSS) in the adult eyes of animals carrying *PRE* fragments next to a *mini-w+* reporter. Fragments from the *vg pPRE* and *dPRE* are indicated in Fig 2, and inserted in the same phiC31 landing site. Reduced *mini-w+* expression in animals homozygous for an insertion indicates silencing mediated by a *PRE*-containing fragment.

To test the silencing effects of these *PRE*s, we created transgenes including these two regions, and integrated these at the same landing site in the Drosophila genome. We then tested if these transgenes induce pairing-sensitive silencing (PSS), a diagnostic feature of *PRE*s where a transgene reporter gene is silenced in homozygous animals [23]. Previous studies showed that a *dPRE*-containing transgene will cause PSS [24], and a similar transgene with the 1 kb *dPRE* region integrated at the landing site also causes PSS (**Fig 3**). We found that a transgene with the 300 bp *pPRE* region also shows strong PSS, demonstrating that the *pPRE* is also a silencing element. These *PRE*s can interact with each other and cause silencing, as animals heterozygous for a *pPRE* transgene in one landing site and a *dPRE* transgene on the homolog also show PSS (**Fig 3**).

CUT&RUN for Polycomb defined a 200 bp segment where Polycomb binds near the *vg* promoter (**Fig 2A**). We used high-resolution mapping by native ChIP in Drosophila S2 cells to precisely define binding sites for three juxtaposed Polycomb-bound sites, one of which is also bound by the Pleiohomeotic (PHO) transcription factor (**S1 Fig**). Deletion of one of these peaks from the *pPRE* transgene alleviates PSS (**Fig 3**), thus this sequence is required for reporter silencing. We analyzed the *dPRE* similarly. We found that while a transgene including the *dPRE* induces PSS, deletion of the Polycomb-bound site within the *dPRE* alleviates this silencing (**Fig 3**). We conclude that the Polycomb-bound sites in both the *pPRE* and *dPRE* elements are required for transgene silencing.

### Silencing of the *vg* domain requires H3K27 methylation

**Fig 4:**
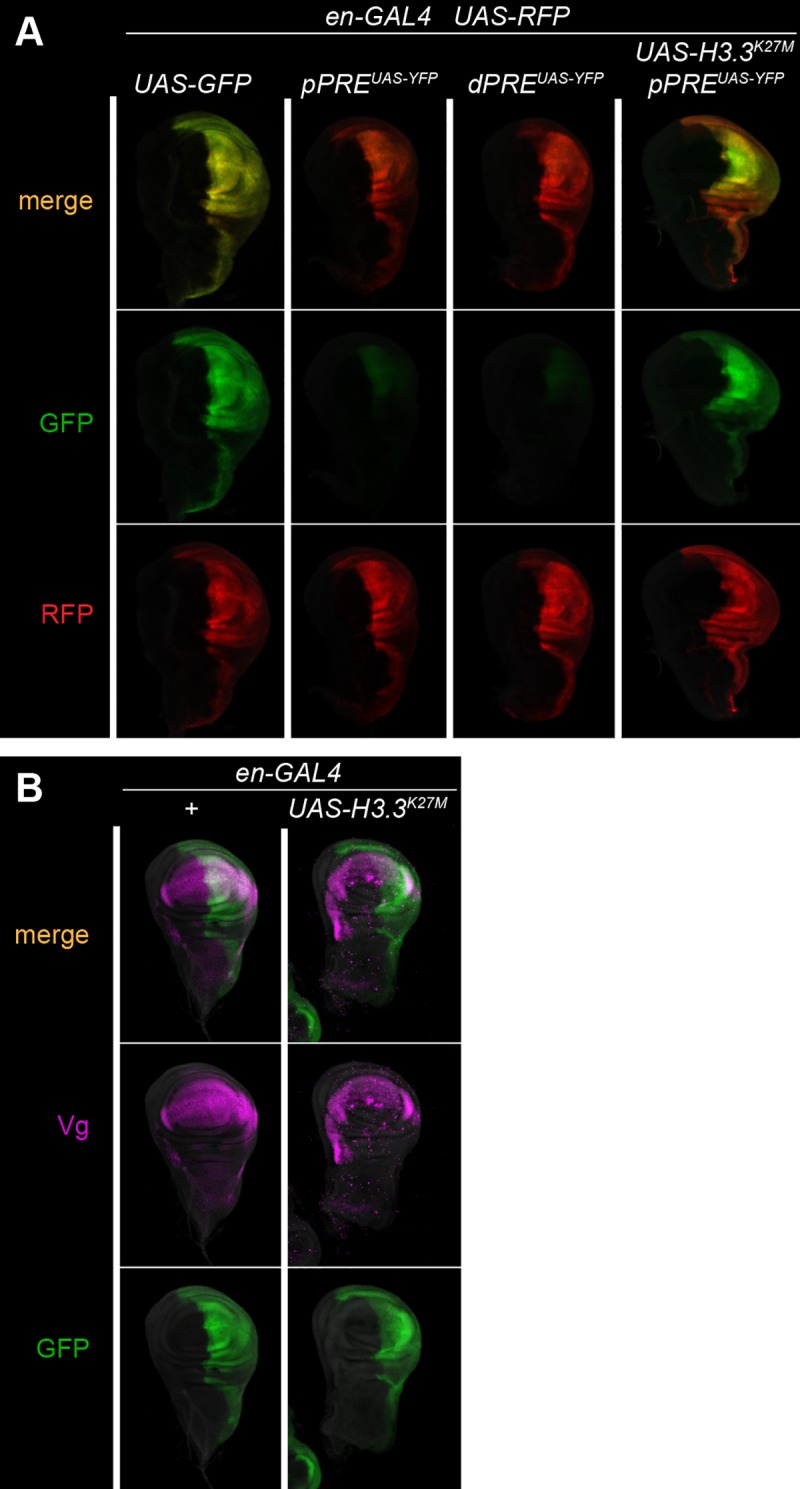
The *vestigial* domain mediate silencing of reporter genes. (A) Fluorescent protein expression in wing imaginal discs. Each animal carries the *en-GAL4* driver, and a *UAS-RFP* control reporter (red). Expression of *UAS-GFP* and *UAS-YFP* is in green. Control insertions outside of Polycomb-regulated domains shows high expression of GFP where *en-GAL4* is expressed. *UAS-YFP* insertions near the *vg pPRE* or *dPRE* show strong silencing, except in the wing pouch where *vg* is normally expressed. Silencing is eliminated by expression of a mutant H3.3^K27M^ histone in the posterior half of the wing disc. (B) The *vg* gene is not derepressed by the mutant H3.3^K27M^ histone. Expression of GAL4 was visualized with *UAS-GFP* (green), and the Vg protein by antibody staining (magenta).

To measure silencing at the endogenous *vg* locus, we integrated reporter genes by gene-targeting near the *pPRE* and near the *dPRE*. The promoter of the *engrailed* gene is active in the posterior half of the wing imaginal disc [25], including part of the expression domain of *vg* in the wing pouch. We used an *engrailed-GAL4* (*en-GAL4*) transgene [26] to drive expression of GAL4-dependent *UAS-YFP* and *UAS-RFP* reporters in the wing disc. Control reporter gene insertions produce RFP and YFP throughout the posterior half of the wing disc (**Fig 4A**). In contrast, *UAS-YFP* reporters inserted in the *vg* domain are silenced throughout most of the wing disc, with reduced expression only within the posterior part of the wing pouch (**Fig 4A**). Insertions near the *dPRE* show similar reduced expression in the wing pouch and silencing in the rest of the wing disc. Thus the *vg* domain appears to be packaged in repressed chromatin in most of the wing disc, but in derepressed chromatin in the wing pouch (**Fig 2A**).

We confirmed that silencing in the wing disc is mediated by chromatin by expressing a dominant-negative H3.3^K27M^ mutant histone to reduce chromatin levels of H3K27me3 [27] in the posterior half of the wing disc. Indeed, expression of the mutant histone derepressed the GFP reporter gene throughout the posterior half of the wing disc (**Fig 4A**). This demonstrates that the *vg* domain is in two chromatin states in the wing imaginal disc: a silenced configuration, and a derepressed configuration in wing pouch cells where *vg* is normally expressed. However, expression of the mutant histone does not derepress expression of the *vg* gene itself (**Fig 4B**). Thus, the silenced configuration appears to only affect the inserted reporter gene.

### The *pPRE* is required for *vestigial* expression

To define the function of the *PRE*s near the *vg* gene, we deleted each element from the endogenous locus (see Methods). Precise breakpoints for each of the recovered deletions were determined by Sanger sequencing, and tested against each other and against previously characterized *vg* alleles (**Fig 2B and 2C**; **S1 Table**; **S2 Table**). We used the *vg*^*nw*^ allele—which deletes part of the coding region of the gene—as a null allele. Ectopic expression of *vg* converts legs and eyes into wing-like structures [28]; thus deletion of silencing elements should derepress the *vg* gene and transform non-wing tissues. However, we observed no such transformations in animals homozygous for deletion of the *pPRE* or of the *dPRE*. Deletion of both *PRE*s from the *vg* domain did not transform non-wing tissues. Thus, there appears to be no role for Polycomb silencing in limiting *vg* expression.

**Fig 5:**
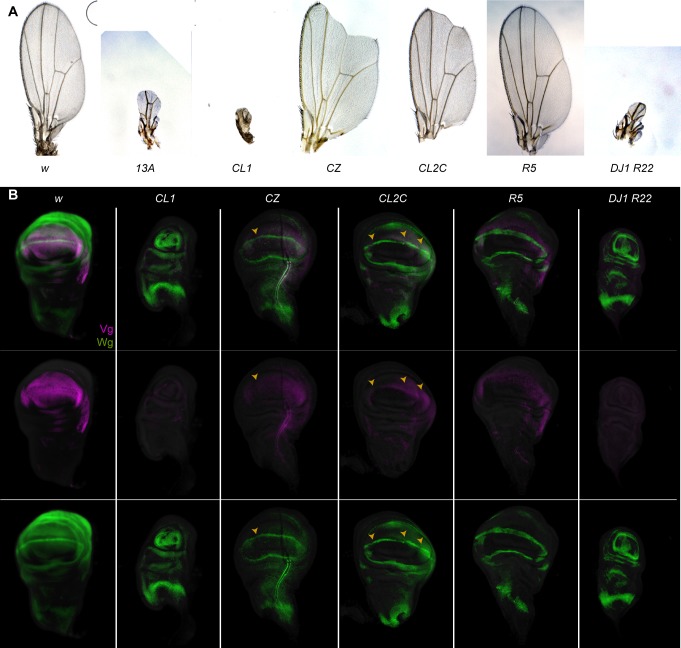
The *pPRE* and Polycomb are required for *vestigial* activation. (A) Wings from adults with the indicated genotypes. New alleles were heterozygous with the *vg*^*nw*^ null allele to show expression of each allele. (B) Wing imaginal discs stained with anti-Vg (magenta) and anti-Wg (green) antisera. Wing discs from control GFP-marked larvae and mutant were dissected, stained, and imaged together for quantitative measurements of antibody staining. In wildtype, a ring of Wg circles the wing pouch, and a stripe of Wg across the wing pouch marks the future adult wing blade margin (gaps indicated by a yellow arrowhead). Vg protein is produced throughout the wing pouch. In *vg*^*CL1*^*/vg*^*nw*^ animals no Vg protein is detected, the wing pouch is greatly reduced, and the future wing margin is missing. In *vg*^*CZ*^*/vg*^*nw*^ animals expression of Vg is reduced, and gaps are apparent in the wing margin Wg stripe, anticipating the wing margin notches in adults of this genotype. Both Vg and Wg expression patterns appear normal in the *dPRE* deletion *vg*^*R5*^. Finally, the double *PRE* deletion *vg*^*DJ1 R22*^ resembles the single *pPRE* deletion, with loss of Vg expression and of the margin stripe of Wg.

Surprisingly, we found that deletions of the *pPRE* reduce expression of the *vg* gene. Wing development is sensitive to the amount of Vg protein, and reduction in *vg* expression results in progressive notching and deletion of the wing. We found that animals carrying the *vg*^*CL1*^ or *vg*^*13A*^ deletions are viable, but have severely reduced wings (**Fig 5A**). A smaller deletion (*vg*^*CZ*^) which deletes only one of the Polycomb-binding sites within the *pPRE* has a more limited effect. The *vg*^*CZ*^ allele gives no phenotype as a homozygote, but in combination with a null allele adults have notched wings which characteristic of weak *vg* alleles. We also recovered a similarly weak allele (*vg*^*CL2C*^) that is a single-base pair C-to-T substitution in the *pPRE*. This substitution lies precisely at the center of the major Polycomb-bound site, in a sequence similar to consensus motifs for Sp1 transcription factors which direct Polycomb binding [29]. Each of these mutations are recessive and viable alleles, and thus are distinct from deletions of the *vg* promoter, which are recessive lethal alleles [22]. Thus, the *pPRE* appears to positively contribute to *vg* expression. In contrast, animals with a deletion of the *dPRE* (the *vg*^*R5*^ allele) are viable with normal wings, either as a homozygote (**Fig 5A**). Animals lacking both *PRE*s are wingless like the *pPRE* single mutant (**Fig 5A**).

The adult wing blade differentiates from the pouch of the larval wing imaginal disc where *vg* is expressed, and we therefore imaged Vestigial protein in wing discs of larvae. Animals carrying the *pPRE* deletion *vg*^*CL1*^ have small wing discs where the wing pouch is reduced, no Vestigial protein is detectable, and the central stripe of Wingless (Wg) signalling ligand that marks the edge of the future wing blade is absent (**Fig 5B**). The smaller *pPRE* deletion *vg*^*CZ*^ and the point mutant *vg*^*CL2C*^ have more limited effects: Vg is produced, but with occasional gaps in wing discs (**Fig 5B, yellow arrowheads**). These gaps are often associated with gaps in the central Wg stripe, consistent with the notching of adult wings. In contrast, the *dPRE* deletion *vg*^*R5*^ has no extra staining or defects of Vg, and the central Wg stripe is continuous. Finally, animals carrying both the *pPRE* and *dPRE* double deletions have small wing discs similar to the single *pPRE* deletion, with no Vg protein detected in wing discs (**Fig 5B**). We conclude that the *pPRE* is required for expression of the *vg* gene in wing discs, but the *dPRE* is not.

**Fig 6:**
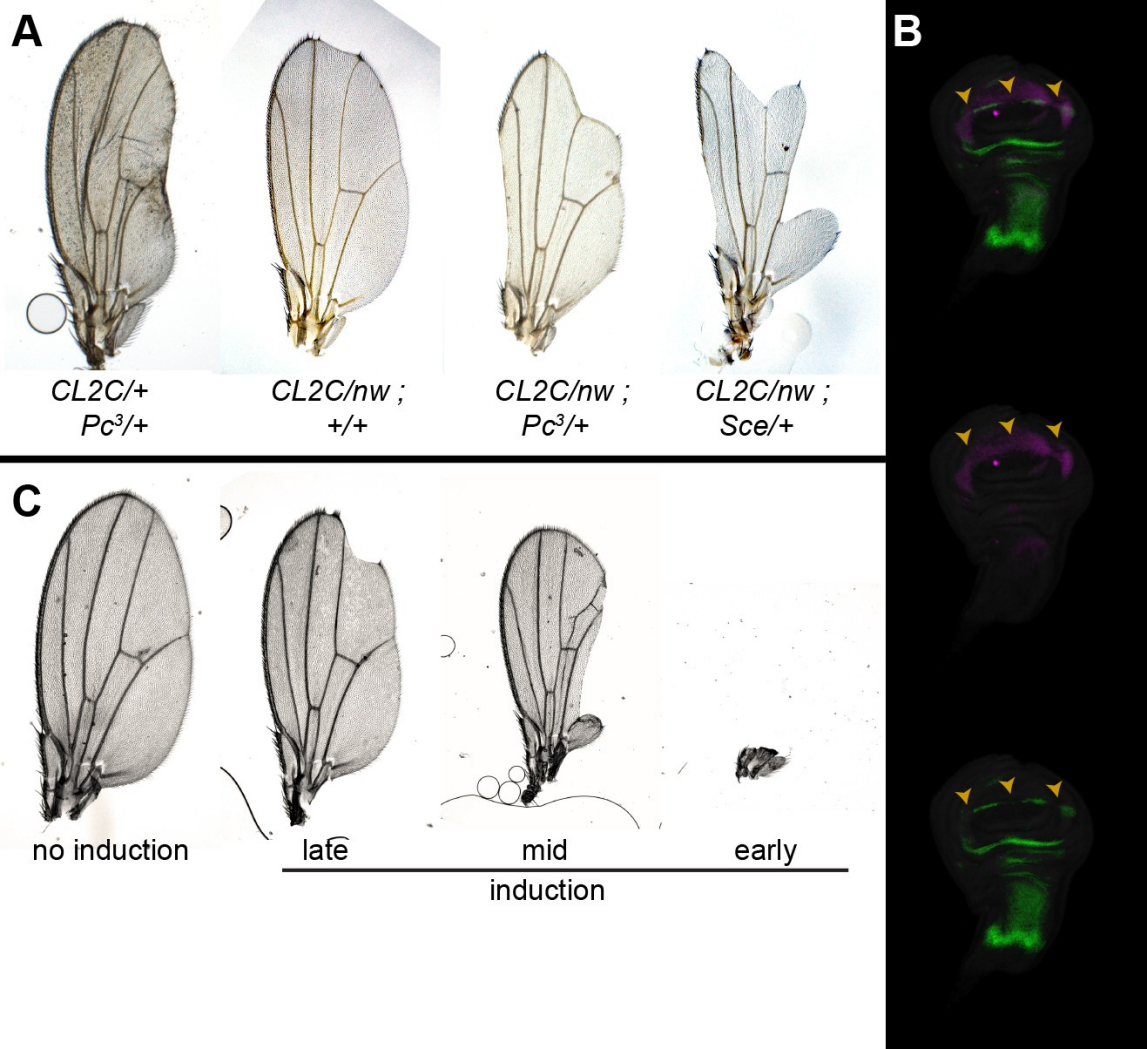
The *pPRE* is required in active cells. (A) Wings from adults with the indicated genotypes. The *pPRE* substitution mutation *vg*^*CL2C*^ is a recessive mutation, but reduction of Polycomb results in bowed and crumpled wings. *Polycomb* or *Sce* mutations enhance the wing defects of *vg*^*CL2C*^*/vg*^*nw*^ animals. (B) Wing imaginal discs from CL2C/nw; Pc3/+ larvae stained with anti-Vg (magenta) and anti-Wg (green) antisera. Gaps in the Vg and Wg patterns at the future wing margin are indicated by a yellow arrowhead. (C) Wing morphologies after *FLP* induction during embryonic and larval development. Most wings show no defects in morphology. Occasional adults that experienced FLP induction during development show notches in the wing margin (induced in late larvae), deletion of portions of the wing (induced in early larvae), or complete loss of the wing (induced in embryos).

We then tested if Polycomb factors are required at the *pPRE* for *vg* expression. The *vg*^*CL2C*^ basepair substitution is a recessive allele, and animals heterozygous for this mutation have no phenotype. However, in combination with a heterozygous *Pc*^*3*^ allele, *vg*^*CL2C*^*/+* animals have deformed wings (**Fig 6A**). The wings of *vg*^*CL2C*^/*vg*^*nw*^; *Pc*^*3*^/*+* animals have more enhanced wing notching, while control *Pc*^*3*^*/+* siblings have normal wings (**Fig 6A; Table 1**). Wing discs from *vg*^*CL2C*^/*vg*^*nw*^; *Pc*^*3*^/*+* larvae show gaps in Vg staining and gaps in the central Wg stripe, consistent with the adult notching (**Fig 6B**). Similarly, mutations in the RING1b homolog *Sce* enhances the phenotype of *vg*^*CL2C*^/*vg*^*nw*^ animals (**Fig 6A**). These effects suggest that PRC1 components bound at the promoter in active cells (**Fig 1D**) positively influence *vg* expression. Finally, combining Polycomb mutations with the *vg*^*R5*^ deletion show no wing defects (**Table 1**), demonstrating that the genetic interaction between Polycomb and the *pPRE* allele is specific.

**Table 1:** Number of progeny with wing notching phenotypes with *vg* and *Pc* mutations.

Genotype	Genotype wing notching class^a^						
	0	1	2	3	4	5	6
***CL2C/nw***	0	23	0	0	0	0	0
***CL2C/nw; Pc***^***3***^***/3***	3	0	10	45	0	0	0
***CL2C/CyO***	6	0	0	0	0	0	0
***CL2C/CyO; Pc***^***3***^***/3***	53	0	0	0	0	0	0
***R5/nw***	29	0	0	0	0	0	0
***R5/nw; Pc***^***3***^***/3***	32	0	0	0	0	0	0
***R5/CyO***	35	0	0	0	0	0	0
***R5/CyO; Pc***^***3***^***/3***	55	0	0	0	0	0	0

^**a**^ Adults were scored in six categories based on wing area and wing margin scalloping from full wildtype wings (0) to complete elimination (6) of the wing.

Regulatory structures are present in the 5’UTRs of some transcripts. However, it is unlikely that the mutations we created affect an mRNA function, because they coincide precisely with the chromatin features of the *pPRE*. Further, the single-base pair mutation *vg*^*CL2C*^ is enhanced by Polycomb mutations, supporting the idea that it affects the function of the chromatin element, not an mRNA function. It is unusual for PRC1 to be implicated in transcriptional activity, but there are examples. In one case in the mouse midbrain, Polycomb is required to bring enhancers to the *meis2* gene promoter before *meis2* is expressed, but then is not required after induction [30]. To test if the *vg pPRE* is similarly required before activation of the *vg* gene or if the *pPRE* is required in cells expressing *vg*, we generated cells homozygous for a *pPRE* deletion from heterozygous cells by *FLP* recombinase-mediated mitotic recombination at different times in development [31]. The *pPRE/+* heterozygous animals have no wing defects, but *FLP* expression produces animals with a range of defects in the wing blade, ranging from notches in the wing margin to complete loss of one wing (**Fig 6C**), implying that *pPRE* mutant clones lose *vg* expression whenever they are induced. Together, these results indicate that the *pPRE* is continually required to maintain expression of the *vg* gene.

### The *distal PRE* is required for chromatin methylation

**Fig 7:**
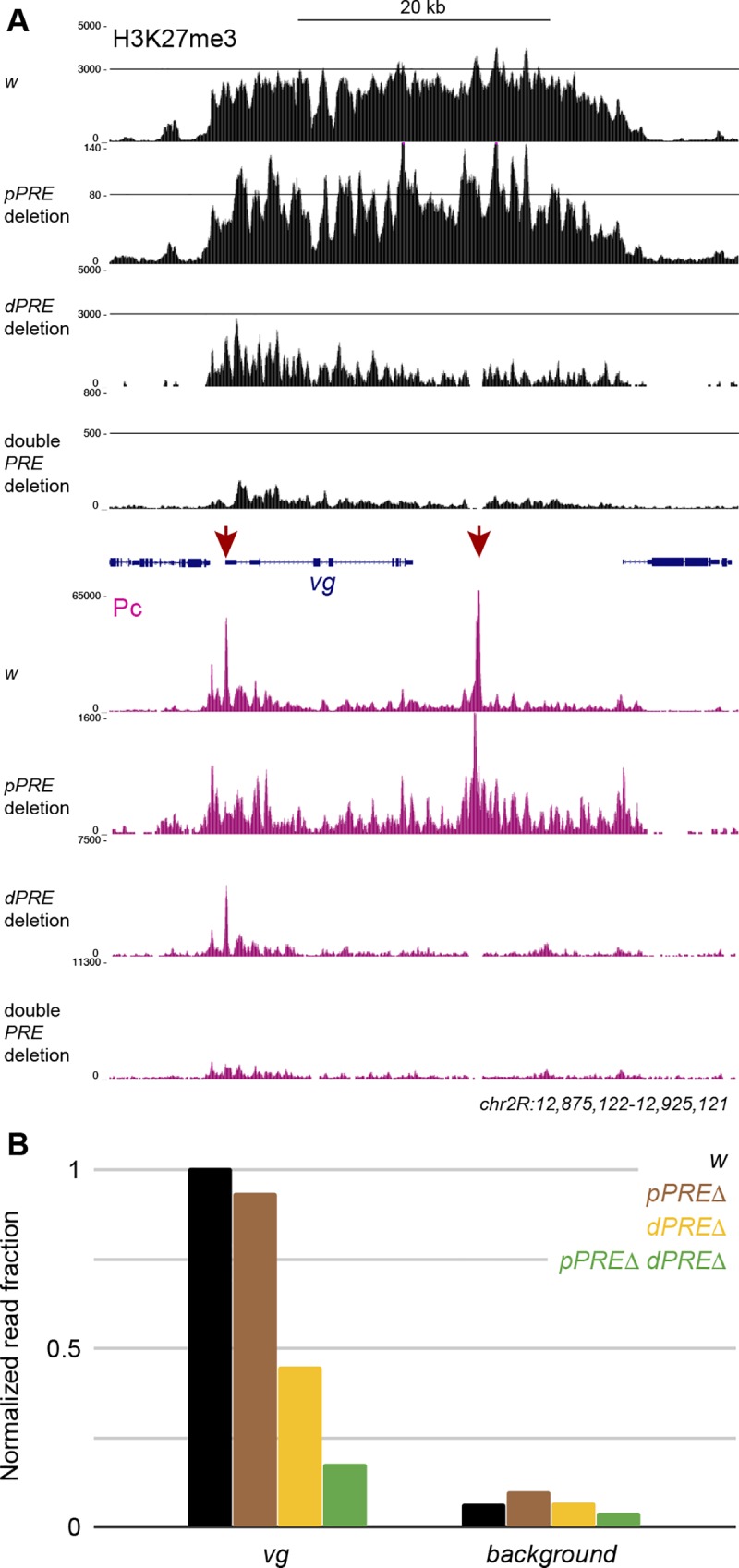
Histone methylation of the *vestigial* domain is reduced in *PRE* mutants. (A) Larval brains from wildtype animals and from *PRE* mutants profiled for H3K27me3 (black) and Polycomb (magenta). The two *PRE*s of the *vg* domain are indicated (red). The display range is set to the maximum signal in the *ANTP-C* domain from the same samples to show quantitative changes in profiling. (B) Normalized mean read counts across the *vg* domain in larval brains. A randomly chosen region outside of annotated domains is used as the genomic background in each sample. Mean read counts in each region were normalized by dividing by the mean read counts at the gsb gene, which is silenced in larval brains.

In transgenes, a *PRE* is required to nucleate and maintain a Polycomb-regulated domain by recruiting PRC1 and PRC2 complexes [6,7]. We tested if histone methylation of the *vg* domain depends on the *pPRE* or on the *dPRE*. We profiled the chromatin of wildtype and *PRE* deletion mutants in larval brains, where the *vg* gene is not active. H3K27me3 levels are high across the *vg* domain in wildtype larval brains (**Fig 7**). However, there is no reduction in H3K27me3 across the *vg* domain In animals lacking the *pPRE*. In contrast, histone methylation is reduced to ~45% of wildtype levels in animals lacking the *dPRE* (**Fig 7**). Finally, histone methylation is reduced to ~20% wildtype levels when both *PRE*s deleted. These results indicate that the *dPRE* is predominantly responsible for histone methylation of the vg domain, although the *pPRE* can also contribute to domain methylation. Notably, the residual methylation across the *vg* domain when both *PRE*s are deleted remains higher than background levels of H3K27me3 in the genome. The amount of residual methylation is similar to that in cells where the *vg* gene is active, suggesting that this is the minimal level of H3K27 methylation of this domain.

It is possible that minor or cryptic *PRE*s in a domain may direct histone methylation when major *PRE*s are deleted [11]. We therefore profiled Polycomb binding in larval brains from wildtype and *PRE* deletion mutants, normalizing landscapes to peak heights in the *ANTP-C* domain (**Fig 7A, bottom**). Deletion of either the *pPRE* or the *dPRE* eliminates only its peak of Polycomb binding. We observe no Polycomb binding when both *PRE*s are deleted, suggesting that there are no alternative or cryptic *PRE*s in the *vg* domain. Thus, the low level of histone methylation across the *vg* domain appears to be independent of Polycomb binding sites.

## Discussion

The concept that Polycomb-repressed chromatin domains are nucleated at short factor-binding regulatory elements (*PRE*s) derives from the phenotypes of deletions within the homeobox clusters in Drosophila, where *PRE*s are required for silencing of these genes [32]. However, it has been difficult to determine if *PRE*s control histone modifications in these domains. While transgenes can confer H3K27me3 modification onto their insertion sites [6,33,34], the effects of deleting *PRE*s from endogenous domains has been more ambiguous. In part these ambiguities may be due to the difficulties of measuring changes in histone modifications with limited samples. Using the more efficient CUT&RUN method, we find that we can produce detailed chromatin profiles from small samples with high sensitivity, allowing characterization of specific tissues in mutant animals.

### *PRE*s are only partially responsible for domain methylation

The developmental gene *vestigial* is contained within chromatin that has the features of a Polycomb chromatin domain, being marked by the H3K27me3 histone modification and silencing inserted reporter transgenes. While the two *PRE*s from this domain both act as silencers in transgenes, our results show that they have distinct roles at the endogenous locus. One *PRE* is primarily responsible for histone methylation of the domain, but has no effect on silencing or expression of the *vg* gene. Previous studies have also suggested that *PRE*s differ in their effects. For example, one of two *PRE*s at the *dachshund* locus also directs methylation of its domain [35]. In contrast, deletions of the known *PRE*s of the *engrailed* locus had no effect on domain methylation [11]. Cryptic *PRE*s or non-coding RNAs have been proposed to direct histone methylation in these situations. However, there we found that no new *PRE*s appear in the *vg* domain, and while non-coding RNAs have been identified near one of the *PRE*s in the *vg* domain [36], these RNAs are deleted in our mutants. Thus, the *vg* domain appears to have a low level of undirected H3K27-methylation. Such domain methylation might be directed by other histone modifications. Active chromatin regions are often marked by H3K27-acetylation, which is antagonistic to H3K27 methylation [37]. Further, in Drosophila cells, the H3K27 methyltransferase E(z) acts globally and dimethylates ~50% of all nucleosomes [38]. Perhaps sequence features of some regions predispose unacetylated regions to accumulate H3K27-trimethylation, and these regions can then reach high levels of H3K27me when *PRE*s are active.

The second *PRE* in the *vg* domain is distinct; while it has little effect on domain methylation, it is required for normal *vg* expression. *PRE* localization near promoters is a common feature of the Drosophila genome [12,39], and are well-positioned to regulate gene activity. Polycomb can silence gene expression by inhibiting transition of RNA polymerase II (RNAPII) to its elongating form, and this is a major step for controlling the expression of developmental genes [40,41]. However, ~1000 active promoters in the Drosophila genome are also bound by Polycomb [12]. While this binding has been suggested to reduce transcriptional output, loss of Polycomb results in both loss of silencing at some genes and decreased transcription at others [39]. In genome-wide studies it has been difficult to determine if downregulation is due to pleiotropic effects or a requirement for Polycomb at some genes, The *vg* gene is the first example of a promoter that requires a *PRE* for expression during development. Positive effects may be mediated by *PRE*s looping together enhancers and promoters [30] as well as silencing elements. The effects of a specific *PRE* may then depend on what regulatory elements it brings to a target promoter. Indeed, *PRE*s have been demonstrated to switch between silencing and activating states [42], and in mammals variant Polycomb complexes have been described that activate developmental genes [43,44]. Our observations that the *vg pPRE* can both silence a reporter gene and promote expression of the endogenous gene suggests that promoters differ in their interactions with *PRE*s, and this may be critical to integrate Polycomb regulation with developmentally-programmed enhancers.

## Methods

### Fly strains

All crosses were performed at 25°C. Transgenes, mutations and chromosomal rearrangements not detailed here are described in Flybase (http://www.flybase.org). The *vg*^*nw*^ allele is a deletion of the last two exons of the *vg* transcript, and so we used this as a standard null allele. New alleles of *vg* produced in this study are described in **S1 Table**.

#### Transgenes

Genomic *PRE*-containing segments were PCR amplified from *Oregon-R* genomic DNA and cloned by Gibson assembly into the split-*mini-w+* vector pKC27mw [23] (a gift from L. Ringrose, Humboldt-Univerität zu Berlin). Deletions within these genomic segments were generated by PCR amplification of the plasmid. The genomic segments in all constructs was verified by sequencing. Plasmids were injected into *y* M[*vas-int*.*Dm*]ZH-2A *w*; *P*[*attP*,*y+*,*w*^*3'*^]VIE-260B embryos by Bestgene Inc (Chino Hills, CA). This line contains two landing sites [45]; integrants at the 25C landing site were used in this study. A line with an integrated plasmid with no genomic fragment was used as a negative control. To produce GAL4 in Vestigial-positive cells, we cloned the *vg* Quadrant enhancer (chr2R:12,986,173–12,896,970 dm6) and GAL4 into pKC27mw, and injected it into *y* M[*vas-int*.*Dm*]ZH-2A *w*; *P*[*attP*,*y+*,*w*^*3'*^]VIE-260B embryos. The integrated construct (named *vgQ-GAL4*.CO) was used to drive UAS-GFP expression in the developing pouch of the wing imaginal disc. Finally, to produce a mutated H3.3K27M protein, we cloned the *UASp* promoter and the *His3*.*3A* ORF into pKC27mw, and then used site-directed mutagenesis PCR to make the K27M mutation. This plasmid was injected into *y* M[*vas-int*.*Dm*]ZH-2A *w*; *P*[*attP*,*y+*,*w*^*3'*^]VIE-260B embryos, and integrants at the 25C site were recovered.

#### *P* mutagenesis

The *vg*^*21-3*^ allele carries a *P* element inserted at the *pPRE* (chr2R:12,884,623 dm6). The *P* element was mobilized by crossing to the *TMS*, *P*[Δ*2–3*]99B transposase source, mosaic males were crossed to *vg*^*nw*^/CyO females, and lines were established from single animals with reduced wing phenotypes. Each mutant was characterized by sequencing an amplicon spanning the *vg*^*21-3*^ insertion site.

#### CRISPR mutagenesis

Guide RNAs to direct Cas9 to the *pPRE* were cloned by overhang PCR into the pU6sgshort plasmid [46] (a gift from N. Perrimon, Harvard Medical School, Boston). Two plasmids with guide RNAs to either side of the *pPRE* were co-injected into *y M[nos-Cas9*.*P*, *w+]*ZH-2A *w* embryos by Bestgene Inc (Chino Hills, CA). These mosaic animals were mated to *w; vg*^*nw*^*/CyOG*, and Cy^+^ progeny with reduced wings were used to establish stocks.

#### Targeted integration and resolution

We used a two-step strategy (**S2 Fig**) to first create a tandem duplication of a deleted *PRE* and a marker gene next to the wildtype *PRE* at the endogenous locus, and then in a second step to delete the wildtype *PRE* and the marker gene, leaving the deleted *PRE* in the locus. To create the tandem duplication, we used CRISPR to induce a double-strand break at a genomic target site, which invades homology on an injected plasmid, integrating it into the chromosome. In the second step, we induced expression of the *ISce-I* site-specific endonuclease to cleave within the tandem duplication (similar to [47]), stimulating recombination between the repeated sequences and resolving to an unmarked locus with the mutant *PRE*.

To do so, we cloned a *UAS-YFP* transgene into pUC19, then cloned genomic segments including the *vestigial pPRE* (1.8 kb) or the *dPRE* (2.9 kb). Deletions within the genomic segments were generated by PCR amplification of the plasmid. Guide RNAs to direct Cas9 to the *pPRE* or to *dPRE* were cloned into pU6sgshort, and co-injected with a plasmid carrying homology on each side of a *PRE* into *y M[nos-Cas9*.*P*, *w+]*ZH-2A *w* or *y w*^*1118*^*; attP2{nos-Cas9}/TM6C*,*Sb Tb* embryos by Bestgene Inc (Chino Hills, CA). Injected mosaic animals were crossed to *w; P*[*GMR-GAL4*]D *P*[*UASRFP*, *w*^*+*^]3, and progeny with YFP-positive eyes were used to establish stocks and then the order of the duplicated segments was determined by PCR. Lines with a tandem duplication in the order (mutant PRE*–UAS-YFP–wildtype *PRE*) were crossed to *y w; P*[*HS-ISce-I*, *v*^*+*^]2B *Sco* / *S*^*2*^*CyO*, and progeny were heat-shocked to express the endonuclease. Mosaic y w / Y; PRE*–UAS-YFP–PRE / P[*HS-ISce-I*, *v*^*+*^]2B *Sco* males were crossed to *w; P*[*GMR-GAL4*]D *P*[*UASRFP*, *w*^*+*^]3, and Sco+ males with no YFP expression were used to establish stocks. Resolution of tandem duplications was confirmed by Sanger sequencing.

To construct a mutant chromosome deleted for both the *pPRE* and the *dPRE*, we injected two plasmids encoding guide RNAs targeting the *pPRE* into a dPRE*–UAS-YFP–dPRE tandem duplication stock, and recovered a *pPRE* deletion by screening for reduced wings. We then resolved the tandem duplication to delete the *dPRE*, producing a double mutant chromosome.

#### Generation of mutant clones

Vials with *P*[*hsFLP*, *ry*^*+*^]1 *y w; P*[*FRT-w*^*hs*^*-FRT*]G13 *vg*^*CL1*^ */ P*[*FRT-w*^*hs*^*-FRT*]G13 larvae were heat-shocked at 37° for 1 hour in a water bath to induce *FLP* expression and mitotic recombination. Adults were examined for defects in wing morphology.

#### Imaging wing imaginal discs

We dissected wing imaginal discs from late 3rd instar larvae and fixed them for 20 minutes in 4% formaldehyde/PBST (PBS with 0.1% triton-X100).

For quantitative detection of proteins in wing discs, tissues from GFP-positive controls and mutant animals were dissected and incubated with antibodies in the same well, Fixed issues were blocked with 10% goat serum/PBST, and incubated with anti-Vg (1:100 dilution, a gift from S Carroll, University of Wisconsin-Madison) and anti-Wg (1:200 dilution, clone 4D4, Developmental Studies Hybridoma Bank) antiserum at 4° overnight, and with fluorescently-labeled secondary antibodies (1:200 dilution, Jackson ImmunoResearch). All tissues were stained with 0.5 μg/mL DAPI/PBS, mounted in 80% glycerol on slides, and imaged by epifluorescence on an EVOS FL Auto 2 inverted microscope (Thermo Fisher Scientific) with a 10X objective.

#### Imaging adult wings

Wings were wetted in ethanol and mounted in 80% glycerol on slides, and photographed using a Sony digital camera mounted on a Nikon SMZ1500 stereomicroscope or with a 4X objective on an EVOS FL Auto 2 inverted microscope (Thermo Fisher Scientific).

#### Imaging adult eyes

Adult heads were mounted on steel pins, immersed in mineral oil, and imaged using a Sony digital camera mounted on a Nikon SMZ1500 stereomicroscope.

### Biological material for chromatin profiling

#### Drosophila cell culture

S2 cells were grown to mid-log-phase in HyClone Insect SFX media (GE). We used 1x10^6^ cells for each chromatin profiling experiment.

#### Intact larval brains and wing imaginal discs

We dissected tissues from 3rd instar larvae in Insect SFX media. We used ~10 larval brains and 20 wing imaginal discs for each chromatin profiling experiment.

#### Isolated wing pouch cells

We dissociated and isolated cells from the vestigial-expressing portion of wing imaginal discs as described [48], with the following modifications. Approximately 200 wing imaginal discs from *vgQ-GAL4*.CO *P*[*UAS-GFP*, *w*^*+*^]T2 3rd instar larvae were dissected in SFX media and dissociated in Accutase (Sigma) for 1 hr at room temperature with occasional agitation. Accutase was blocked with 0.5 volumes of FBS. The solution was passed through a 40 μm filter and sorted for GFP fluorescence using 488 nm excitation and 530/30 nm emission filters on an BD Aria II Flow Cytometer using a flow rate of 3 (~20 μL/min) and a 100 μm nozzle. We used ~1x10^4^ cells for each chromatin profiling experiment.

### Chromatin profiling

#### CUT&RUN

We used an immuno-tethered strategy for profiling histone modifications and Polycomb binding in Drosophila cells. The CUT&RUN method uses an antibody to a specific chromatin epitope to tether a protein A fused to micrococcal nuclease (p-AMNase) at chromosomal binding sites within permeabilized cells [14]. The nuclease is activated by the addition of calcium, and cleaves DNA around binding sites. Cleaved DNA is isolated and subjected to paired-end Illumina sequencing to map the distribution of the chromatin epitope. We used primary antibodies to histone H3 trimethylation at lysine-27 (clone C36B11, Cell Signalling Technology, Inc), and to Drosophila Polycomb (a gift from G Cavalli, Université de Montpellier) We used a low-cell number version of CUT&RUN [15] for Drosophila S2 cells, intact larval tissues, and cells collected after FACS with the following modifications. S2 cells and cells collected after FACS were bound to BioMag Plus Concanavalin-A-conjugated magnetic beads (ConA beads, Polysciences, Inc), permeabilized in dbe+ buffer (20 mM HEPES pH 7.5, 150 mM NaCl, 0.9 mM spermidine, 2 mM EDTA, 0.1% BSA, 0.05% digitonin with Roche cOmplete protease inhibitor), cleaved in WashCa+ buffer buffer (20 mM HEPES pH 7.5, 150 mM NaCl, 0.9 mM spermidine, 0.1% BSA, 2 mM CaCl_2_ with Roche cOmplete protease inhibitor) at 0° for 30 minutes, and then DNA was recovered as described [15]. In some cases we collected S2 cells by centrifugation and cleaved chromatin for 5’ at room temperature. For intact larval brains and wing discs, we transferred tissues between buffers in in the wells of glass dissection dishes by pipetting with low-retention tips. Tissues were incubated with dbe+ buffer, incubated in primary antibodies overnight at 4° with gentle rocking, and incubated in pAMN/dbe+ for 1 hour at room temperature. After washing, tissues were transferred to eppendorfs with chilled WashCa+ buffer and incubated at 0° for 30’ for DNA cleavage, then stopped, treated with RNase A for 30 minutes at 37°, and spun at 16,000 g for 5 minutes at 4° to separate supernatant and pellet fractions, and processed as described [15]. In all experiments we added 0.3 pg of yeast fragmented DNA as a spike-in normalization control for library preparation and sequencing [14]. Two replicates were performed for each experiment. In some experiments we coated dissected tissues with ConA beads and moved them between solutions using a magnet, and recovered cleaved DNA with Ampure XP beads (Beckman Coulter) immediately after protease treatment. A step-by-step protocol is posted (https://protocols.io/private/D6B0AD2DC1431A513994A2A05AC59CDA).

#### Library preparation, sequencing, and data processing

Libraries were prepared as described [14], with 14 cycles of PCR with 10 second extensions for enrichment of short DNA fragments. Libraries were sequenced for 25 cycles in paired-end mode on the Illumina HiSeq 2500 platform at the Fred Hutchinson Cancer Research Center Genomics Shared Resource. Paired-end reads were mapped to release r6.30 of the *D*. *melanogaster* genome obtained from FlyBase using Bowtie2, and to the yeast genome (SacCer3) for spike-in normalization, normalizing fly profiles to the number of yeast reads. Track screenshots were produced using the UCSC Genome browser (http://genome.ucsc.edu) [49]. To compare histone methylation profiles between samples, H3K27me3 domains were annotated on the *X*, *2*, and *3* chromosomes in wildtype larval brain samples and the mean read counts in each domain were measured (**Supplementary Information**).

#### Data accession codes

Sequencing data are available in GEO at National Center for Biotechnology Information (GSE121028).

## Supporting information

S1 Fig Native ChIP mapping of chromatin factors at the *vestigial PRE*s in Drosophila S2 cells. Correspondence of CUT&RUN mapping of *PRE* features to bound transcription factors detected by ORGANIC native-ChIP (native-ChIP profiling reported in [13]).
(PDF)

S2 Fig Two-step deletion of regulatory elements. The scheme is designed to eliminate a genomic segment (white box) through a series of targeted recombination events. In (1), the YFP donor plasmid with an internally-deleted homology region is co-injected with a CRISPR gRNA plasmid into embryos expressing Cas9. Cleavage of the chromosome by Cas9 stimulates recombination (blue lines) between the donor plasmid and the chromosome. (2) Flies with the resulting tandem duplication in the chromosome are recovered by crossing injected animals to a GAL4 driver line and screening progeny for YFP-expressing progeny. In (3), flies with the tandem duplication are crossed to animals with an heat-shock-inducible *ISce-I* gene, and the progeny are heat-shocked. These animals are crossed to a GAL4 driver line, and progeny that do not express YFP are recovered as potential intra-chromosomal recombination events that have reduced the tandem duplication (4). Deletion of the regulatory element is confirmed by PCR amplification of the homology region and sequencing.
(PDF)

S1 Table New mutations of the *vestigial* gene. (DOCX)

S2 Table Complementation of new *vestigial* alleles. (DOCX)

S3 Table Mean read counts of H3K27me3 CUT&RUN in Polycomb-regulated domains. (DOCX)
