# Supplementary figures and images for: Separate Polycomb Response Elements control chromatin state and activation of the vestigial gene
Source: PLoS Genet. 2019 Aug 19;15(8):e1007877. doi: 10.1371/journal.pgen.1007877 (PMC6730940; doi:10.1371/journal.pgen.1007877)

Supplementary Figure 1

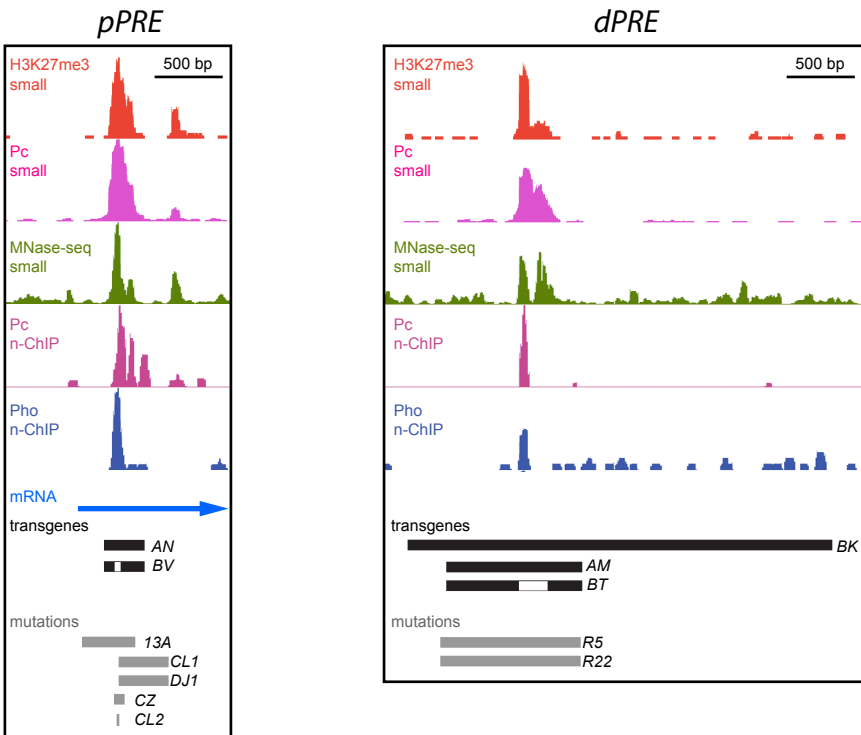

Supplement: S1 Fig — Correspondence of CUT&RUN mapping of PRE features to bound transcription factors detected by ORGANIC native-ChIP (native-ChIP profiling reported in [13]). (PDF) [file pgen.1007877.s001.pdf]

1

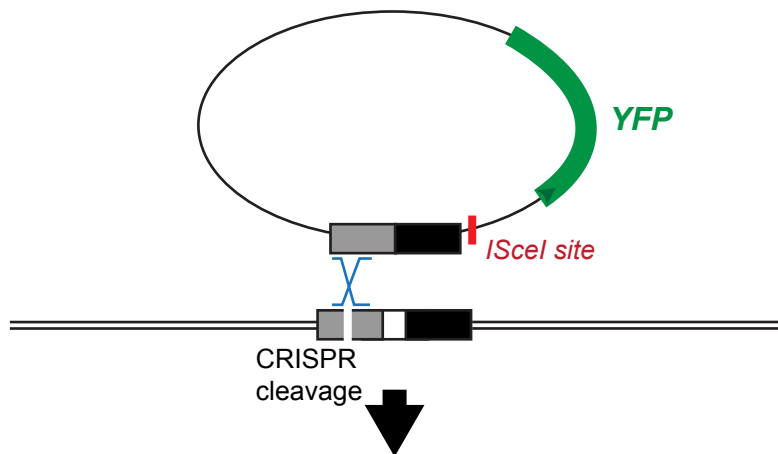

2

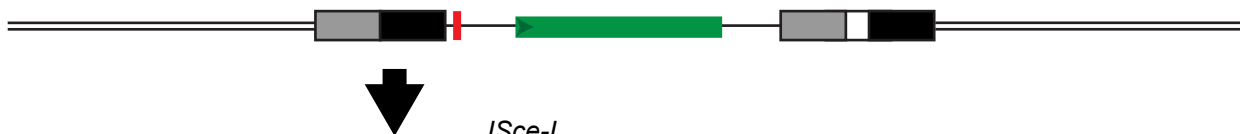

3

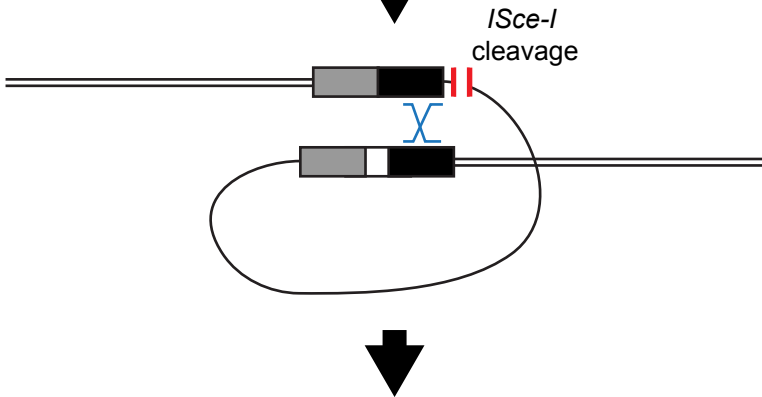

4

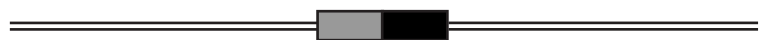

Supplement: S2 Fig — The scheme is designed to eliminate a genomic segment (white box) through a series of targeted recombination events. In (1), the YFP donor plasmid with an internally-deleted homology region is co-injected with a CRISPR gRNA plasmid into embryos expressing Cas9. Cleavage of the chromosome by Cas9 stimulates recombination (blue lines) between the donor plasmid and the chromosome. (2) Flies with the resulting tandem duplication in the chromosome are recovered by crossing injected animals to a GAL4 driver line and screening progeny for YFP-expressing progeny. In (3), flies with the tandem duplication are crossed to animals with an heat-shock-inducible ISce-I gene, and the progeny are heat-shocked. These animals are crossed to a GAL4 driver line, and progeny that do not express YFP are recovered as potential intra-chromosomal recombination events that have reduced the tandem duplication (4). Deletion of the regulatory element is confirmed by PCR amplification of the homology region and sequencing. (PDF) [file pgen.1007877.s002.pdf]
