# Supplementary material for: Separate Polycomb Response Elements control chromatin state and activation of the vestigial gene
Source: PLoS Genet. 2019 Aug 19;15(8):e1007877. doi: 10.1371/journal.pgen.1007877 (PMC6730940; doi:10.1371/journal.pgen.1007877)
Supplement: S1 Table — (DOCX) [file pgen.1007877.s003.docx]

**Supplementary Table 1. New mutations of the *vestigial* gene.**

***mutation position from TSS^1^ method of induction structure***

| 13A |  | 32 | – | 423 |  |  | *P* mobilization |  | deletion and insertion of TCAT |
| --- | --- | --- | --- | --- | --- | --- | --- | --- | --- |
| CL1 |  | 303 | – | 667 |  |  | CRISPR |  | deletion |
| CZ2 |  | 263 | – | 344 |  |  | CRISPR, ISceI cleavage |  | deletion |
| CL2C |  |  |  | 296 |  |  | CRISPR |  | C -> T substitution |
| DJ1 |  | 303 | – | 667 |  |  | CRISPR |  | deletion |
| R5 |  | 19614 | – | 20640 |  |  | CRISPR, ISceI cleavage |  | deletion |
| R22 |  | 19614 | – | 20640 |  |  | CRISPR, ISceI cleavage |  | deletion |
|  |  |  |  |  |  |  |  |  |  |

^1^The *vg* Transcriptional Start Site (TSS) is located at 2R:12,884,200 dm6.
