# Supplementary material for: Separate Polycomb Response Elements control chromatin state and activation of the vestigial gene
Source: PLoS Genet. 2019 Aug 19;15(8):e1007877. doi: 10.1371/journal.pgen.1007877 (PMC6730940; doi:10.1371/journal.pgen.1007877)
Supplement: S2 Table — (DOCX) [file pgen.1007877.s004.docx]

**Supplementary Table 2. Complementation^a^ of new *vestigial* alleles.**

| ***Df(2R)***  ***vgD*** | ***Df(2R)***  ***Exel8056*** | ***vg^13A^*** | ***vg^CL1^*** | ***vg^CZ^*** | ***vg^CL2C^*** | ***vg^nw^*** | ***vg^R5^*** | ***vg^DJ1R22^*** |  |
| --- | --- | --- | --- | --- | --- | --- | --- | --- | --- |
| lethal | lethal | 6 | 5 | 1 | 4 | lethal | 0 | 5 | ***Df(2R)vgD*** |
|  | lethal | 5 | 5 | 1 | 3 | 6 | 0 | 6 | ***Df(2R)Exel8056*** |
|  |  | 5 | 5 | 1 | 0 | 5 | 0 | 5 | ***vg^13A^*** |
|  |  |  | 5 | 0 | 0 | 5 | 0 | 5 | ***vg^CL1^*** |
|  |  |  |  | 0 | 0 | 2 | 0 | 0 | ***vg^CZ^*** |
|  |  |  |  |  | 0-1 | 3 | 0 | 1 | ***vg^CL2C^*** |
|  |  |  |  |  |  | lethal | 0 | 5 | ***vg^nw^*** |
|  |  |  |  |  |  |  | 0 | 0 | ***vg^R5^*** |
|  |  |  |  |  |  |  |  | 5 | ***vg^DJ1R22^*** |

**^a^** Allelic combinations were scored in six categories based on adult wing area and wing margin scalloping from full wildtype wings (0) to complete elimination (6) of the wing, or as lethal combinations.
