# Supplementary material for: Separate Polycomb Response Elements control chromatin state and activation of the vestigial gene
Source: PLoS Genet. 2019 Aug 19;15(8):e1007877. doi: 10.1371/journal.pgen.1007877 (PMC6730940; doi:10.1371/journal.pgen.1007877)
Supplement: S3 Table — (DOCX) [file pgen.1007877.s005.docx]

**Supplementary Table 3. Mean read counts in H3K27me3 in Polycomb-regulated domains.**

**wt pPREΔ dPREΔ pPREΔ wt wt**

**length includes dPREΔ wing**

**Domain (bp) gene brain brain brain brain wing pouch**

| chrX:4,370,994-4,531,058 |  | 160,065 | bi | 1521.3 | 33.7 | 928.1 | 156.0 | 461.5 | 73.3 |
| --- | --- | --- | --- | --- | --- | --- | --- | --- | --- |
| chrX:4,553,804-4,630,106 |  | 76,303 | peb | 1813.7 | 47.4 | 1055.1 | 221.5 | 727.2 | 176.3 |
| chrX:5,528,783-5,613,165 |  | 84,383 | Vsx1 | 2040.4 | 47.2 | 1360.1 | 202.7 | 787.6 | 232.6 |
| chrX:5,992,179-6,035,492 |  | 43,314 | mab-21 | 1130.7 | 40.4 | 644.6 | 157.1 | 205.9 | 43.4 |
| chrX:7,109,284-7,281,980 |  | 172,697 | CG9650 | 998.1 | 27.1 | 785.7 | 114.1 | 447.5 | 125.4 |
| chrX:7,487,140-7,679,574 |  | 192,435 | ct | 1070.1 | 33.8 | 700.5 | 74.0 | 508.7 | 147.4 |
| chrX:8,606,412-8,676,929 |  | 70,518 | oc | 1870.6 | 53.3 | 1271.6 | 255.7 | 818.8 | 307.2 |
| chrX:8,721,959-8,838,074 |  | 116,116 | Lim1 | 1391.6 | 36.3 | 891.8 | 147.2 | 630.7 | 194.9 |
| chrX:9,282,401-9,317,695 |  | 35,295 | lz | 1307.2 | 54.5 | 911.1 | 166.2 | 612.7 | 174.8 |
| chrX:9,684,482-9,778,956 |  | 94,475 | Sp1 | 1384.9 | 41.3 | 982.8 | 171.4 | 646.9 | 212.7 |
| chrX:16,104,082-16,264,617 |  | 160,536 | disco | 1093.7 | 33.4 | 895.8 | 143.8 | 550.8 | 216.2 |
| chrX:17,305,401-17,418,446 |  | 113,046 | B-H2 | 1438.7 | 42.3 | 794.8 | 189.1 | 562.4 | 199.5 |
| chrX:17,750,912-17,820,780 |  | 69,869 | Ods-H | 1713.4 | 45.0 | 1284.3 | 218.1 | 747.6 | 283.4 |
| chrX:18,239,572-18,320,578 |  | 81,007 | upd3 | 1237.1 | 36.3 | 859.5 | 93.5 | 478.2 | 120.2 |
| chrX:19,531,912-19,560,302 |  | 28,391 | CG32532 | 904.2 | 52.2 | 468.8 | 180.1 | 358.9 | 166.6 |
| chr2L:358,619-400,588 |  | 41,970 | al | 1311.2 | 48.3 | 925.5 | 114.1 | 590.5 | 172.3 |
| chr2L:490,319-535,760 |  | 45,442 | ush | 1205.0 | 40.6 | 778.4 | 84.4 | 532.3 | 220.6 |
| chr2L:577,030-611,394 |  | 34,365 | Gsc | 1951.3 | 68.5 | 1473.2 | 269.1 | 800.1 | 280.8 |
| chr2L:1,633,938-1,652,192 |  | 18,255 | chinmo | 1251.9 | 44.2 | 874.2 | 73.6 | 586.7 | 181.4 |
| chr2L:1,944,018-1,974,268 |  | 30,251 | erm | 1881.6 | 70.6 | 1397.8 | 253.8 | 748.3 | 229.5 |
| chr2L:2,423,536-2,489,125 |  | 65,590 | dpp | 1062.4 | 40.5 | 745.0 | 64.6 | 521.6 | 138.5 |
| chr2L:3,530,788-3,618,981 |  | 88,194 | drm | 2049.4 | 63.3 | 1369.3 | 286.9 | 761.1 | 280.9 |
| chr2L:3,657,600-3,705,195 |  | 47,596 | Drgx | 958.5 | 41.3 | 664.2 | 179.9 | 290.0 | 106.7 |
| chr2L:3,813,566-3,862,839 |  | 49,274 | slp1 | 1755.7 | 57.6 | 1260.0 | 263.9 | 839.6 | 296.2 |
| chr2L:5,365,764-5,517,366 |  | 151,603 | H15 | 1900.4 | 53.2 | 1386.7 | 224.2 | 788.1 | 316.9 |
| chr2L:6,527,307-6,557,745 |  | 30,439 | eya | 1365.3 | 59.5 | 1100.7 | 179.9 | 672.2 | 198.8 |
| chr2L:6,802,779-6,852,351 |  | 49,573 | sens-2 | 1504.5 | 66.6 | 1268.7 | 263.3 | 482.6 | 139.5 |
| chr2L:7,281,730-7,342,902 |  | 61,173 | wg | 1406.4 | 54.5 | 1096.5 | 139.9 | 579.8 | 191.4 |
| chr2L:7,350,675-7,382,504 |  | 31,830 | Wnt-10 | 1193.8 | 42.3 | 564.4 | 88.4 | 482.3 | 168.7 |
| chr2L:8,740,783-8,888,885 |  | 148,103 | SoxN | 1377.6 | 40.0 | 1023.4 | 135.0 | 626.0 | 208.6 |
| chr2L:9,576,465-9,614,042 |  | 37,578 | gcm | 1834.7 | 78.5 | 1149.9 | 275.4 | 733.6 | 184.3 |
| chr2L:11,285,849-11,494,327 |  | 208,479 | salr | 1490.5 | 47.3 | 1116.2 | 159.9 | 337.1 | 83.4 |
| chr2L:12,074,606-12,093,615 |  | 19,010 | prd | 1334.7 | 52.4 | 880.4 | 105.3 | 659.1 | 212.3 |
| chr2L:12,543,054-12,689,826 |  | 146,773 | nub | 2011.0 | 64.2 | 1669.9 | 297.7 | 465.2 | 97.1 |
| chr2L:15,067,945-15,113,508 |  | 45,564 | CG15269 | 1083.4 | 55.5 | 1186.5 | 90.9 | 521.2 | 203.2 |
| chr2L:15,270,892-15,499,671 |  | 228,780 | wor | 727.9 | 31.5 | 468.4 | 67.9 | 227.9 | 52.5 |
| chr2L:16,350,295-16,491,778 |  | 141,484 | dac | 1007.4 | 38.8 | 661.7 | 126.6 | 471.9 | 199.2 |
| chr2L:18,713,939-18,831,344 |  | 117,406 | ham | 1334.8 | 46.2 | 889.8 | 188.8 | 502.4 | 182.0 |
| chr2L:20,106,714-20,121,327 |  | 14,614 | Kua | 1131.9 | 84.1 | 1314.0 | 96.1 | 550.3 | 169.8 |
| chr2L:20,766,673-20,787,552 |  | 20,880 | cad | 2243.3 | 95.9 | 2309.3 | 482.1 | 864.0 | 388.5 |
| chr2L:21,763,026-21,981,079 |  | 218,054 | tsh | 1446.9 | 54.9 | 982.8 | 189.3 | 418.3 | 247.5 |
| chr2L:21,991,736-22,043,873 |  | 52,138 | tio | 1172.4 | 46.7 | 957.1 | 113.0 | 386.8 | 209.6 |
| chr2R:5,705,816-5,754,698 |  | 48,883 | ap | 1625.1 | 73.2 | 1211.8 | 315.7 | 338.9 | 136.4 |
| chr2R:7,407,138-7,440,595 |  | 33,458 | so | 1371.0 | 58.3 | 964.3 | 216.8 | 651.4 | 272.1 |
| chr2R:7,988,855-8,069,659 |  | 80,805 | optix | 1475.5 | 59.6 | 1497.0 | 261.7 | 601.9 | 271.0 |
| chr2R:9,190,953-9,204,921 |  | 13,969 | unpg | 1464.8 | 92.1 | 1577.8 | 323.4 | 561.0 | 289.9 |
| chr2R:9,972,606-9,989,546 |  | 16,941 | eve | 1937.1 | 70.6 | 1737.6 | 277.0 | 789.4 | 287.4 |
| chr2R:11,462,123-11,579,310 |  | 117,188 | inv-en | 1971.9 | 58.3 | 1598.4 | 323.0 | 541.7 | 202.7 |
| chr2R:12,882,316-12,917,933 |  | 35,618 | vg | 2142.4 | 68.9 | 757.0 | 48.7 | 439.2 | 81.0 |
| chr2R:12,936,113-13,043,488 |  | 107,376 | Psc | 1675.5 | 56.8 | 1056.1 | 202.9 | 570.8 | 244.8 |
| chr2R:14,189,394-14,215,355 |  | 25,962 | Sox15 | 1383.0 | 53.0 | 1052.2 | 106.2 | 478.0 | 213.9 |
| chr2R:14,434,110-14,467,412 |  | 33,303 | Oaz | 1849.3 | 62.0 | 1367.2 | 232.5 | 737.4 | 251.3 |
| chr2R:14,772,698-14,846,099 |  | 73,402 | kn | 1650.9 | 58.0 | 1091.0 | 184.8 | 559.7 | 134.0 |
| chr2R:15,802,809-15,856,895 |  | 54,087 | Poxn | 1307.2 | 58.4 | 1025.5 | 218.6 | 481.1 | 155.3 |
| chr2R:19,242,521-19,281,754 |  | 39,234 | rib | 1889.7 | 77.5 | 1708.2 | 324.7 | 744.5 | 331.6 |
| chr2R:20,884,037-20,977,193 |  | 93,157 | Rx | 1865.8 | 60.7 | 1499.7 | 247.3 | 730.8 | 257.9 |
| chr2R:22,216,824-22,298,664 |  | 81,841 | dve | 1658.6 | 55.0 | 1061.1 | 162.5 | 692.0 | 139.0 |
| chr2R:23,617,494-23,652,487 |  | 34,994 | retn | 2204.3 | 84.1 | 1761.8 | 347.8 | 867.1 | 288.0 |
| chr2R:24,337,205-24,391,786 |  | 54,582 | bs | 944.7 | 45.4 | 931.3 | 63.6 | 347.5 | 61.8 |
| chr2R:24,796,993-24,870,390 |  | 73,398 | Dll | 1483.3 | 46.1 | 1129.4 | 155.8 | 662.7 | 147.9 |
| chr2R:25,029,745-25,078,164 |  | 48,420 | gsb | 2126.6 | 73.6 | 1680.7 | 276.7 | 939.4 | 377.1 |
| chr2R:25,082,737-25,141,690 |  | 58,954 | lov | 1733.3 | 49.8 | 954.1 | 234.2 | 638.6 | 148.1 |
| chr2R:25,159,451-25,254,664 |  | 95,214 | Kr | 2072.5 | 73.5 | 1471.4 | 348.8 | 705.6 | 323.3 |
| chr3L:365,967-435,163 |  | 69,197 | trh | 1876.6 | 62.2 | 1390.8 | 234.4 | 600.1 | 124.6 |
| chr3L:1,095,827-1,110,256 |  | 14,430 | bab1 | 1060.8 | 46.9 | 922.0 | 67.6 | 447.1 | 90.9 |
| chr3L:1,163,468-1,192,232 |  | 28,765 | bab2 | 1000.9 | 50.3 | 976.9 | 91.2 | 413.3 | 135.6 |
| chr3L:1,352,906-1,485,484 |  | 132,579 | ru | 802.5 | 25.5 | 461.8 | 37.2 | 375.6 | 129.8 |
| chr3L:3,616,469-3,714,664 |  | 98,196 | dar1 | 751.6 | 40.0 | 593.4 | 122.2 | 285.9 | 101.8 |
| chr3L:3,841,258-3,894,616 |  | 53,359 | Awh | 1369.1 | 60.2 | 979.7 | 188.3 | 548.7 | 198.7 |
| chr3L:5,814,838-5,895,922 |  | 81,085 | vn | 629.9 | 31.4 | 430.2 | 103.6 | 258.3 | 91.7 |
| chr3L:6,094,713-6,125,134 |  | 30,422 | Ets65A | 459.2 | 27.9 | 267.2 | 148.4 | 25.9 | 17.3 |
| chr3L:6,763,829-6,943,428 |  | 179,600 | vv1 | 1037.2 | 41.1 | 721.9 | 113.5 | 228.7 | 52.3 |
| chr3L:6,981,181-6,994,405 |  | 13,225 | bin1 | 1293.6 | 51.6 | 1008.9 | 174.5 | 634.6 | 203.0 |
| chr3L:7,938,292-7,970,785 |  | 32,494 | exex | 1475.7 | 71.0 | 1065.8 | 276.3 | 475.0 | 168.0 |
| chr3L:8,635,349-8,718,912 |  | 83,564 | h | 523.1 | 26.0 | 240.4 | 70.3 | 146.4 | 38.9 |
| chr3L:9,002,460-9,047,332 |  | 44,873 | Doc1 | 1718.6 | 52.9 | 1467.4 | 175.6 | 801.2 | 232.2 |
| chr3L:10,898,797-11,039,963 |  | 141,167 | klu | 593.6 | 25.0 | 522.2 | 51.5 | 246.1 | 90.6 |
| chr3L:11,917,838-11,960,416 |  | 42,579 | byn | 1009.4 | 53.1 | 870.7 | 89.8 | 536.7 | 209.0 |
| chr3L:12,277,172-12,336,449 |  | 59,278 | Lmx1a | 481.2 | 23.6 | 212.6 | 122.7 | 37.9 | 17.0 |
| chr3L:12,418,168-12,471,793 |  | 53,626 | toe | 1475.4 | 55.5 | 1152.6 | 217.4 | 528.0 | 226.3 |
| chr3L:12,541,180-12,733,196 |  | 192,017 | IroC | 1760.4 | 54.0 | 1229.1 | 236.7 | 478.4 | 208.4 |
| chr3L:13,390,196-13,411,189 |  | 20,994 | sens | 1175.3 | 56.2 | 768.3 | 112.7 | 510.4 | 158.5 |
| chr3L:14,109,308-14,191,219 |  | 81,912 | D | 1917.3 | 63.6 | 1316.3 | 254.0 | 773.7 | 303.3 |
| chr3L:14,563,084-14,625,177 |  | 62,094 | HGTX | 1832.3 | 74.8 | 1670.3 | 308.6 | 674.3 | 342.4 |
| chr3L:15,014,456-15,058,355 |  | 43,900 | ind | 332.1 | 29.4 | 251.7 | 115.1 | 26.6 | 16.5 |
| chr3L:19,642,510-19,685,169 |  | 42,600 | tey | 1842.5 | 71.7 | 1554.0 | 230.6 | 637.5 | 257.3 |
| chr3L:20,540,590-20,710,845 |  | 170,256 | kni | 1146.2 | 53.7 | 942.7 | 141.7 | 420.2 | 148.7 |
| chr3L:21,449,974-21,484,389 |  | 34,416 | croc | 2196.3 | 84.9 | 1713.2 | 351.1 | 840.5 | 365.7 |
| chr3L:21,576,695-21,613,549 |  | 36,855 | Tfap-2 | 1322.3 | 63.9 | 1084.2 | 292.4 | 549.4 | 250.4 |
| chr3L:24,717,931-24,749,682 |  | 31,752 | scro | 1195.4 | 107.3 | 1311.4 | 372.3 | 406.3 | 216.1 |
| chr3R:4,820,578-4,906,530 |  | 85,953 | opa | 2162.3 | 61.2 | 1872.3 | 329.9 | 862.5 | 392.9 |
| chr3R:6,656,490-7,068,009 |  | 411,520 | ANTP-C | 2263.1 | 54.6 | 1916.0 | 307.3 | 724.3 | 380.1 |
| chr3R:7,293,146-7,346,750 |  | 53,605 | rn | 1254.3 | 54.5 | 986.9 | 103.6 | 380.3 | 87.4 |
| chr3R:7,925,683-7,973,715 |  | 48,033 | dsx | 1221.3 | 54.5 | 915.0 | 110.8 | 556.4 | 244.1 |
| chr3R:8,129,291-8,234,037 |  | 104,747 | grn | 1764.4 | 51.5 | 1399.4 | 241.9 | 553.2 | 281.5 |
| chr3R:8,269,884-8,301,346 |  | 31,463 | ato | 1469.6 | 53.1 | 780.3 | 172.3 | 565.6 | 165.2 |
| chr3R:8,316,793-8,339,787 |  | 22,995 | Poxm | 1888.8 | 63.8 | 1454.5 | 185.0 | 841.4 | 320.3 |
| chr3R:8,678,757-8,712,777 |  | 34,021 | hb | 683.6 | 34.6 | 432.3 | 225.0 | 38.0 | 18.7 |
| chr3R:10,360,673-10,378,068 |  | 17,396 | Rfx | 384.1 | 24.6 | 208.1 | 119.8 | 32.3 | 16.8 |
| chr3R:10,416,151-10,675,353 |  | 259,203 | hth | 982.4 | 34.5 | 645.3 | 98.5 | 251.8 | 181.4 |
| chr3R:11,248,503-11,313,057 |  | 64,555 | CR31386 | 627.7 | 29.7 | 349.7 | 162.6 | 32.6 | 17.4 |
| chr3R:11,353,295-11,379,214 |  | 25,920 | pros | 1390.9 | 48.8 | 1148.7 | 124.8 | 670.7 | 234.2 |
| chr3R:12,222,027-12,347,864 |  | 125,838 | svp | 1178.9 | 51.1 | 984.5 | 155.9 | 422.3 | 126.5 |
| chr3R:13,057,031-13,078,532 |  | 21,502 | sim | 1934.6 | 84.8 | 1416.1 | 305.4 | 766.2 | 325.7 |
| chr3R:13,839,727-13,957,893 |  | 118,167 | ems | 1748.7 | 55.0 | 1237.0 | 281.3 | 574.3 | 233.5 |
| chr3R:15,984,403-16,040,986 |  | 56,584 | pnr | 2341.1 | 75.7 | 1790.5 | 343.0 | 855.7 | 392.8 |
| chr3R:16,359,114-16,432,387 |  | 73,274 | ss | 1778.5 | 56.1 | 1420.2 | 298.9 | 588.1 | 257.9 |
| chr3R:16,656,887-16,989,359 |  | 332,473 | BX-C | 3025.8 | 58.9 | 2658.2 | 424.0 | 1029.9 | 514.4 |
| chr3R:17,546,474-17,575,343 |  | 28,870 | Hmx | 1098.3 | 44.4 | 702.4 | 249.0 | 239.3 | 73.1 |
| chr3R:18,040,713-18,160,799 |  | 120,087 | sr | 991.8 | 41.8 | 723.5 | 67.2 | 411.7 | 137.8 |
| chr3R:18,368,272-18,388,396 |  | 20,125 | gl | 869.7 | 57.2 | 529.5 | 186.2 | 386.1 | 96.2 |
| chr3R:21,376,594-21,572,495 |  | 195,902 | lbl | 2077.2 | 54.8 | 1679.1 | 290.3 | 828.2 | 350.1 |
| chr3R:23,128,738-23,152,218 |  | 23,481 | hh | 1117.6 | 48.6 | 841.3 | 116.1 | 524.9 | 163.1 |
| chr3R:23,190,687-23,215,167 |  | 24,481 | cnc | 2257.1 | 85.8 | 1771.6 | 333.4 | 885.2 | 372.3 |
| chr3R:23,285,926-23,332,192 |  | 46,267 | pnt | 1236.1 | 53.2 | 892.1 | 165.3 | 617.3 | 244.1 |
| chr3R:25,076,519-25,097,873 |  | 21,355 | fd96Ca | 1737.1 | 82.7 | 1684.7 | 435.0 | 807.9 | 409.2 |
| chr3R:25,131,407-25,194,016 |  | 62,610 | lobo | 729.1 | 36.3 | 471.5 | 60.8 | 444.1 | 166.3 |
| chr3R:26,062,808-26,108,381 |  | 45,574 | dysf | 749.9 | 35.0 | 626.0 | 49.1 | 433.0 | 171.3 |
| chr3R:27,168,955-27,227,317 |  | 58,363 | Ser | 561.5 | 32.0 | 382.7 | 84.1 | 120.3 | 32.2 |
| chr3R:28,544,455-28,597,983 |  | 53,529 | fkh | 1942.5 | 69.3 | 1364.7 | 252.2 | 746.8 | 224.7 |
| chr3R:29,503,586-29,592,521 |  | 88,936 | Dr | 2301.4 | 65.5 | 1853.3 | 280.3 | 483.7 | 176.4 |
| chr3R:29,682,047-29,723,806 |  | 41,760 | dmrt99B | 1620.2 | 48.5 | 1159.9 | 201.1 | 601.1 | 150.9 |
| chr3R:30,601,129-30,789,227 |  | 188,099 | zfh1 | 1069.8 | 36.1 | 1087.4 | 79.7 | 503.0 | 227.2 |
| chr3R:30,888,604-30,946,792 |  | 58,189 | Ptx1 | 1363.5 | 58.5 | 1062.1 | 219.2 | 429.5 | 104.0 |
| chr3R:31,060,906-31,086,567 |  | 25,662 | Sox100B | 1526.6 | 59.4 | 1290.2 | 90.1 | 667.6 | 219.8 |
|  |  |  |  |  |  |  |  |  |  |
| **background regions** |  |  |  |  |  |  |  |  |  |
| chr2R:5,770001-6,270,000 |  | 500,000 | EcR | 134.6 | 7.3 | 116.5 | 11.0 | 34.9 | 18.5 |
| chr2R:16,300,076-16,800,075 |  | 500,000 | DAT | 103.5 | 7.8 | 114.0 | 14.5 | 32.1 | 18.8 |
| chr2R:10,933,910-11,433,909 |  | 500,000 | shn | 127.5 | 8.8 | 113.4 | 12.3 | 36.0 | 21.5 |
| chr2R:5,440,978-5,690,977 |  | 250,000 | Fis1 | 86.9 | 6.0 | 101.5 | 12.3 | 31.8 | 23.9 |
| chr2R:8,375,564-8,625,563 |  | 250,000 | mtt | 109.9 | 7.5 | 105.4 | 15.7 | 31.1 | 17.2 |
| chr2R:10,375,564-10,625,563 |  | 250,000 | lola | 66.8 | 6.2 | 125.7 | 12.3 | 19.5 | 21.6 |
| chr2R:5,111,842-5,236,841 |  | 125,000 | Nipped-A | 55.3 | 5.6 | 95.0 | 13.8 | 19.9 | 18.1 |
| chr2R:6,111,842-6,236,841 |  | 125,000 | Ptr | 93.3 | 6.3 | 82.9 | 8.9 | 28.3 | 16.6 |
| chr2R:24,111,842-24,236,841 |  | 125,000 | spag | 102.5 | 8.1 | 90.3 | 13.6 | 35.1 | 22.3 |
| chr2R:5,111,840-5,174,339 |  | 62,500 | gus | 31.0 | 5.8 | 79.8 | 10.8 | 16.6 | 17.0 |
| chr2R:8,114,378-8,176,877 |  | 62,500 | kermit | 86.8 | 5.9 | 79.6 | 7.5 | 22.9 | 15.5 |
| chr2R:16,114,378-16,176,877 |  | 62,500 | Lbk | 76.6 | 7.1 | 83.2 | 8.1 | 30.6 | 29.3 |
| chr2R:6,032,376-6,063,625 |  | 31,250 | Tbce | 84.9 | 6.8 | 86.7 | 10.9 | 28.5 | 15.0 |
| chr2R:16,069,171-16,100,420 |  | 31,250 | Dg | 66.4 | 6.3 | 99.1 | 8.6 | 22.8 | 14.5 |
| chr2R:14,017,761-14,049,010 |  | 31,250 | mam | 62.7 | 6.4 | 89.2 | 8.3 | 25.4 | 18.9 |
| chr2R:7,000,652-7,016,276 |  | 15,625 | Tsp42Ec | 75.3 | 5.8 | 85.2 | 6.8 | 20.3 | 12.4 |
